# A national system for monitoring the performance of hospitals in Ethiopia

**DOI:** 10.2471/BLT.14.151399

**Published:** 2015-08-21

**Authors:** Zahirah McNatt, Erika Linnander, Abraham Endeshaw, Dawit Tatek, David Conteh, Elizabeth H Bradley

**Affiliations:** a Yale School of Public Health, 60 College Street, PO Box 208034, New Haven, CT 06520-8034, United States of America.; b Ethiopian Federal Ministry of Health, Addis Ababa, Ethiopia.; c Clinton Health Access Initiative, Addis Ababa, Ethiopia.

## Abstract

Many countries struggle to develop and implement strategies to monitor hospitals nationally. The challenge is particularly acute in low-income countries where resources for measurement and reporting are scarce. We examined the experience of developing and implementing a national system for monitoring the performance of 130 government hospitals in Ethiopia. Using participatory observation, we found that the monitoring system resulted in more consistent hospital reporting of performance data to regional health bureaus and the federal government, increased transparency about hospital performance and the development of multiple quality-improvement projects. The development and implementation of the system, which required technical and political investment and support, would not have been possible without strong hospital-level management capacity. Thorough assessment of the health sector’s readiness to change and desire to prioritize hospital quality can be helpful in the early stages of design and implementation. This assessment may include interviews with key informants, collection of data about health facilities and human resources and discussion with academic partners. Aligning partners and donors with the government’s vision for quality improvement can enhance acceptability and political support. Such alignment can enable resources to be focused strategically towards one national effort – rather than be diluted across dozens of potentially competing projects. Initial stages benefit from having modest goals and the flexibility for continuous modification and improvement, through active engagement with all stakeholders.

## Résumé

De nombreux pays s'efforcent d'élaborer et de mettre en œuvre des stratégies pour contrôler les hôpitaux à l'échelle nationale. C'est un défi de taille, en particulier pour les pays à faible revenu, qui disposent de peu de ressources pour effectuer des mesures et des comptes-rendus. Nous avons analysé l'expérience qui a consisté à élaborer et mettre en œuvre un système national de contrôle des performances de 130 hôpitaux publics en Éthiopie. Des observations participatives ont révélé que ce système de contrôle a entraîné une communication plus systématique des données sur les performances aux bureaux régionaux de la santé et au gouvernement fédéral de la part des hôpitaux, a amélioré la transparence concernant les performances de ces derniers et a permis d'élaborer plusieurs projets d'amélioration de la qualité. L'élaboration et la mise en œuvre de ce système, qui ont nécessité des investissements et des soutiens techniques et politiques, n'auraient pas été possibles sans d'importantes capacités de gestion dans les hôpitaux. Il peut être utile, aux premiers stades de la conception et de la mise en œuvre, d'évaluer de manière approfondie l'ouverture au changement du secteur de la santé ainsi que son souhait de placer au premier plan la qualité des services hospitaliers. Cette évaluation peut s'appuyer sur des entretiens avec des informateurs clés, sur la collecte de données au sujet des établissements de santé et des ressources humaines, ou encore sur une discussion avec des partenaires universitaires. Le fait d'associer des partenaires et des donateurs à l'objectif d'amélioration de la qualité visé par le gouvernement peut permettre de renforcer son acceptabilité ainsi que le soutien politique en la matière. Cela peut permettre de faire converger les ressources de façon stratégique vers un même effort national, plutôt que de les éparpiller dans des dizaines de projets potentiellement concurrents. Au cours des premières phases, il est bénéfique d'avoir des objectifs modestes et de faire preuve de souplesse afin de permettre des modifications et une amélioration continues, par le biais d'un engagement actif auprès de l'ensemble des parties prenantes.

## Resumen

Muchos países tienen dificultades para desarrollar e implementar estrategias a nivel nacional para monitorizar los hospitales. El reto es especialmente complicado en países de ingresos bajos donde los recursos para la medición y la notificación son escasos. Se examinó la experiencia a la hora de desarrollar e implementar un sistema nacional para monitorizar el rendimiento de 130 hospitales del gobierno en Etiopía. Haciendo uso de una observación participativa, se observó que el sistema de monitorización se tradujo en una notificación de datos de rendimiento de los hospitales más coherente a las oficinas de salud regionales y al gobierno federal, así como un incremento en la transparencia sobre el rendimiento de los hospitales y el desarrollo de diferentes proyectos para la mejora de la calidad. El desarrollo y la implementación de dicho sistema, que requería de inversiones y soporte tanto técnico como en materia de inversión, no hubieran sido posibles sin una fuerte habilidad de gestión a nivel hospitalario. Una meticulosa valoración de la disposición del sector sanitario a cambiar y del deseo de este a priorizar la calidad hospitalaria podría ser de gran ayuda en las primeras fases del diseño y la implementación. Esta evaluación podría incluir entrevistas con informadores clave, una recopilación de datos sobre instalaciones sanitarias, recursos humanos y debates con asociados del mundo académico. Poner en consonancia la visión del gobierno sobre la mejora de la calidad con los asociados y contribuyentes puede hacer aumentar la aceptación y el apoyo político. Dicho alineamiento puede permitir una focalización estratégica de los recursos respecto a un solo esfuerzo nacional, antes que esparcirlo en docenas de proyectos potencialmente conflictivos. Las fases iniciales son las principales beneficiadas de tener objetivos modestos y flexibilidad para modificar y mejorar de forma continua, a través de un compromiso activo con todos los accionistas.

## ملخص

تناضل دول كثيرة من أجل تطوير وتطبيق استراتيجيات لمراقبة المستشفيات على المستوى المحلي. ويتسم هذا التحدي بالصعوبة وخاصة في الدول منخفضة الدخل حيث تندر الموارد اللازمة للقياس والتسجيل. وبحثنا في إمكانية تنفيذ تجربة تطوير وتطبيق نظام قومي لمراقبة أداء عدد من المستشفيات في إثيوبيا يبلغ 130 مستشفى تابعة للقطاع الحكومي. وتوصلنا باستخدام الملاحظة القائمة على المشاركة إلى أن نظام المراقبة أدى إلى تسجيل المزيد من الاتساق في بيانات الأداء المتعلقة بالمستشفيات لدى مكاتب الصحة الإقليمية والحكومة الفيدرالية، وزيادة الشفافية فيما يتعلق بأداء المستشفيات، وتطوير العديد من المشروعات التي تهدف إلى تحسين مستوى الجودة. ولم يكن تطوير وتطبيق هذا النظام، الذي تطلب استثمار الجهود التقنية والسياسية وتقديم الدعم بشأنها، ممكنًا لولا قوة الإمكانيات الإدارية على مستوى المستشفى. كما أن الاستعداد للتغيير فيما يتعلق بالتقييم الشامل لقطاع الصحة قد يجدي نفعًا في المراحل الأولى للتخطيط والتطبيق. وقد يشمل هذا التقييم بعض المقابلات مع المبلّغين الرئيسيين للمعلومات، وجمع بعض البيانات عن المنشآت الصحية والموارد البشرية، وإجراء بعض المناقشات مع الشركاء الأكاديميين. كما أن التنسيق مع الشركاء والمتبرعين بما يتوافق مع رؤية الحكومة لإجراء التحسينات في مستوى الجودة يمكن أن يحسِّن من مستوى التقبل والدعم السياسي. ويمكن لمثل هذا التنسيق أن يعمل على تركيز الموارد من الناحية الاستراتيجية على بذل الجهد لتحقيق هدف قومي واحد - بدلًا من تشتت الجهد بين العشرات من المشروعات التي من المحتمل أن تتسم بالتنافس فيما بينها. وقد تتحقق الاستفادة في المراحل الأولية نتيجة السعي نحو تحقيق أهداف بسيطة وتوافر المرونة لإتاحة التعديل والتحسين المستمر، وذلك من خلال المشاركة الفعالة لجميع الجهات المعنية.

## 摘要

许多国家正在尽力制定和实施监测全国医院的战略。 这一挑战在测量和报告资源稀缺的低收入国家尤为严峻。 我们研究了埃塞俄比亚建立和实施监测 130 个政府医院绩效的国家体系的经验。 通过参与观察，我们发现，监测体系不仅能够提高医院向地区卫生局和联邦政府报告的绩效数据的一致性和医院绩效的透明度，而且能够促进多个质量改进项目的开发。 监测体系需要技术和政治上的投资与支持，该体系的建立和实施离不开强大的医院管理能力。 因此，全面评估卫生部门和重点改进医院质量的期望在设计和实施的早期阶段有所帮助。 这项评估可能包括采访关键的知情人、收集与卫生设施和人力资源相关的数据，以及与学术合作伙伴展开讨论。 协调合作伙伴、捐赠者和政府对于质量改进的愿景能够提高该体系的公众接受度和政治支持。 此类协调能够将资源战略性集中于一个国家项目，而非分摊到数十个潜在的竞争项目中。 通过所有利益相关方的积极参与，设立切合实际的目标，以灵活的方式不断改良和完善，在初期阶段获益匪浅。

## Резюме

Ряд стран предпринимают значительные усилия для разработки и внедрения стратегии мониторинга работы больниц в национальном масштабе. Проблема стоит особенно остро в странах с низким уровнем дохода, где недостаточно ресурсов для проведения замеров и составления отчетности. Мы исследовали опыт разработки и внедрения национальной системы мониторинга качества работы 130 правительственных больниц в Эфиопии. С помощьюнепосредственного наблюдения мы обнаружили, что система мониторинга привела к улучшению отчетности больниц о своей работе перед региональными отделениями системы здравоохранения и федеральным правительством. Кроме того, показатели качества работы больниц стали более прозрачными и были разработаны проекты по дальнейшему повышению качества. Разработка и внедрение этой системы потребовали технической и политической поддержки, а также значительных вложений и были бы невозможны без сильного руководства над больницами на местах. Тщательная оценка готовности сектора здравоохранения к переменам и желание вывести качество работы больниц на новый уровень могут быть полезны на ранних этапах разработки и внедрения. В оценку могут включаться опросы ключевых информаторов, сбор данных об учреждениях здравоохранения и кадрах, а также обсуждение с участием академических партнеров. Выравнивание статуса партнеров и доноров с точкой зрения правительства, взявшего курс на улучшение качества обслуживания, может обеспечить одобрение и усилить политическую поддержку. Такое выравнивание может обеспечить стратегическую концентрацию ресурсов на усилиях, необходимых для решения единой национальной задачи, вместо распыления их на десятки потенциально конкурирующих проектов. На первых этапах будет полезна постановка довольно скромных целей и готовность к постоянным изменениям и улучшениям, которые достигаются активным вовлечением всех партнеров.

## Introduction

Improvement in the quality of hospital care is a fundamental aspect of health system strengthening^1^^–^^4^ that is directly linked to the service delivery dimension of the World Health Organization (WHO) building blocks of a health system.^5^ While the monitoring of hospital performance is a key ingredient to such improvement,^1^^,^^3^^,^^4^ many countries struggle to develop and implement feasible strategies to monitor hospitals nationally. The challenge is particularly acute in low-income countries where resources for measurement and reporting are scarce.

In the field of global health, research on performance monitoring often focuses broadly on health systems^6^^–^^9^ rather than on hospitals. The literature on the development and implementation of systems for monitoring hospital performance is largely dominated by case studies and reports from high-income countries with national health systems – e.g. Canada^10^ and Denmark,^11^ the United Kingdom of Great Britain and Northern Ireland^12^ and other countries in western Europe.^13^^–^^15^ Although there has also been some relevant research in the United States of America,^10^ it has tended to focus on a narrow set of quality measures in specific populations.^16^^,^^17^ The WHO performance assessment tool for quality improvement in hospitals is a donor-led, externally designed measurement project rather than a country-led, internally developed initiative.^14^^,^^15^ This tool has been applied in only one middle-income country (South Africa).^14^^,^^15^ Most attempts to monitor hospital performance in low-income settings have involved small numbers of facilities and narrowly defined clinical measures of performance.^18^^–^^24^ When creating their accreditation systems for hospitals, both Liberia and Zambia monitored hospital performance for just a year, to collect baseline data.^25^^,^^26^

We could find no peer-reviewed studies done in low-income countries that described the development and sustained implementation of a national system for monitoring hospital performance, based upon a comprehensive set of key performance indicators. We therefore sought to describe the creation and implementation of such a national system in a low-income country. We considered Ethiopia to be a good setting in which to conduct our case study because of recent hospital reform in the country. The reform led to the creation of: (i) the role of hospital chief executive officer – qualified through a master’s degree programme in hospital and health-care administration;^27^^,^^28^ (ii) private wings in hospitals that allowed revenue generation and (iii) hospital governing boards.^28^^,^^29^

The many new government hospitals that were built during the ongoing reform process led to improved hospital access in both rural and urban settings. We describe the development of key performance indicators, the process of monitoring hospital performance relative to these indicators and the trend in performance since 2010, which marked the implementation of Ethiopia’s national system of hospital monitoring. Findings from this case study may be helpful to other low-income countries seeking to elevate the quality of facility-based health care through performance monitoring and accountability.

## Key performance indicators

### Development

We developed performance indicators that were relevant for hospitals and consistent throughout the country. The first indicator developed was the most fundamental – adherence to national guidelines on hospital management. In 2009, Ethiopia partnered with the Clinton Health Access Initiative and the Yale Global Health Leadership Institute to develop national guidelines for the management of hospitals: the Ethiopian Hospital Reform Implementation Guidelines.^30^^,^^31^ These guidelines included 124 hospital management standards, each of which was a statement – e.g. “the hospital conducts a physical inventory of all pharmaceuticals in the store and each dispensing unit at a minimum of once per year.” Hospitals were asked to report quarterly whether each standard was met.

The success of the rollout of Ethiopia’s first attempt to monitor hospital performance, in 2010, was probably the result of simplicity and focus on hospital management. The guidelines leveraged the Ethiopian Federal Ministry of Health’s investment in the training of hospital chief executive officers via the master’s of hospital and health-care administration degree programme.^28^^,^^32^ The guidelines, the associated scoring sheet, the promotion of adherence to the guidelines and the building of management capacity were made integral parts of the two-year programme. The students in the programme were selected by regional health bureaus.^32^ At the time of writing, more than 90% of those who successfully completed the degree programme remain employed in the Ethiopian health-care sector (D Tatek, unpublished observations, 2014).

Given the reality that, in 2009–2010, government hospitals were understaffed, financially limited and often did not have 24-hour access to basic resources such as water and electricity, the ministry of health agreed that, before launching reporting on other aspects of hospital performance, such as efficiency, cost, clinical outcomes and patient experience – government hospitals should be accountable for meeting a set of minimum management standards.

By 2011, 40% of government hospitals were reporting data on their adherence to operational standards – to both the ministry of health and the appropriate regional health bureau. Improvements were already apparent in the establishment of hospital quality committees, drug formularies, pharmacy inventory control systems and a host of other quality-improvement efforts.^33^ Staff from regional health bureaus and development partners visited hospitals to corroborate the reported information and to provide coaching on the operational standards. The environment was poised for the introduction of a more robust monitoring system based upon multiple key performance indicators.

In 2011, the ministry of health negotiated a standardized set of performance indicators, in partnership with the regional health bureaus and with technical assistance as before, from the Clinton Health Access Initiative and the Yale Global Health Leadership Institute. The process of selecting such indicators for the nation’s hospitals was rigorous and included reviewing other country experiences, development of thematic areas and frequent reviews with federal, regional and external stakeholders. Given the need for these indicators to be reliable – and collection of data on them to be feasible^6^ – the ministry of health held sessions with the regional health bureaus to determine which indicators were most important to the bureaus and what human resource training and infrastructure development were needed to enable collection of the corresponding data.

Six months of research and negotiation resulted in the establishment of 36 national indicators for the assessment of hospital performance. These indicators covered 11 aspects of hospital operations: hospital management, outpatient, emergency, inpatient, maternity, referral and pharmacy services, productivity, human resources, finance and patient experience (Table 1). Together, the indicators encompassed measures of operational functioning, clinical quality, financial activity and patient experience.^34^ The ministry of health worked with partners to limit the number of indicators that could potentially create perverse incentives (e.g. mortality rates) and to explain, to hospital and ministry of health staff, the potential unintended effects of indicator measurement.

**Table 1:** Hospital key performance indicators, Ethiopia, 2010

Category, indicator code	Indicator
**Hospital management**	
KPI 1	Proportion of EHRIG operational standards met
**Outpatient services**	
KPI 2	Outpatient attendees
KPI 3	Outpatient attendees seen by private-wing service
KPI 4	Outpatient waiting time to treatment
KPI 5	Outpatients not seen on same day
**Emergency services**	
KPI 6	ED attendees
KPI 7	ED patients triaged within 5 minutes of arrival at ED
KPI 8	ED attendances with stay longer than 24 hours
KPI 9	ED mortality
**Inpatient services**	
KPI 10	Inpatient admissions
KPI 11	Inpatient admissions to private wing
KPI 12	Inpatient mortality
KPI 13	Delay for elective surgical admission
KPI 14	Bed occupancy
KPI 15	Mean length of stay
KPI 16	Incidence of pressure ulcer
KPI 17	Percentage of surgical sites infected
KPI 18	Completeness of inpatient medical records
**Maternity services**	
KPI 19	Deliveries – i.e. live births and stillbirths – attended
KPI 20	Births by surgical, instrumental or assisted vaginal delivery
KPI 21	Institutional maternal mortality
KPI 22	Institutional neonatal deaths within 24 hours of birth
**Referral services**	
KPI 23	Referrals made
KPI 24	Rate of referrals
KPI 25	Emergency referrals, as a proportion of all referrals made
**Pharmacy services**	
KPI 26	Mean stock-out duration of hospital-specific tracer drugs
**Productivity**	
KPI 27	Patient-day equivalents per physician
KPI 28	Patient-day equivalents per nurse or midwife
KPI 29	Major surgeries per surgeon
KPI 30	Major surgeries conducted in private wing
**Human resources**	
KPI 31	Attrition rate among physicians
KPI 32	Staff experience, as a staff satisfaction rating
**Finance**	
KPI 33	Cost per patient-day equivalent
KPI 34	Raised revenue, as a proportion of total operating revenue
KPI 35	Revenue utilization – i.e. the proportion of budget used
**Patient experience**	
KPI 36	Patient experience, as a patient satisfaction rating

ED: emergency department; EHRIG: Ethiopian hospital reform implementation guidelines; KPI: key performance indicator.

The performance indicators included process measures that were directly related to patient outcomes. For example, one indicator – the average time during which stocks of basic drugs were unavailable – highlighted how often inpatients and outpatients were unable to purchase medications and therefore had to remain untreated or source medications from private pharmacies. Another indicator – the percentage of patients triaged within five minutes of arrival in the emergency department *–* was particularly important to all stakeholders as it directly responded to community concerns about mortality and morbidity resulting from delayed treatment.

The success of the development of the indicators was largely due to the simplicity, flexibility and transparency of the process. The number of indicators was kept small and the focus was on measures that could be calculated reasonably easily by hospital staff. The ministry of health required commitment to reporting the 36 national indicators but allowed regional health bureaus to create additional indicators, which stimulated regional ownership. A *National Hospital Performance Monitoring and Improvement Manual*, which outlined each indicator thoroughly and specified precise definitions and data sources, was disseminated through a series of national workshops funded by the United States Centers for Disease Control and Prevention (CDC) and the ministry of health.

### Implementation and monitoring

The implementation of the monitoring system included substantial investments in both human resources and information technology. In terms of human resources, new roles were developed at the hospital level and in the regional health bureaus and ministry of health. Each hospital had several individuals – so-called data owners – who were each dedicated to collecting data on the performance indicators that were relevant to their department. For example, a midwife could be the data owner for neonatal mortality. In addition, each hospital had an indicator collator who worked closely with each data owner and was responsible for the collation of data on all the indicators. Instead of hiring new personnel to undertake these tasks, most hospitals modified the job descriptions of current employees and provided additional short-term, on-site training. Data on the indicators were initially collected on paper forms and then compiled and submitted as spreadsheet computer files. Health and development partners provided technical support for designing data entry and reporting applications.

At bureau and ministry level, the curative and rehabilitation teams and the medical services directorate were dedicated to the performance indicators and hospital operations. These teams were responsible for training hospital data owners and indicator collators, troubleshooting problems with data collection and reporting and synthesizing the hospital-level data into a national database for comparing hospital performance within and across regions (Table 2). The ministry of health used the summary databases during discussions of trends in hospital performance, at quarterly joint sessions of the regional and federal health leadership.

**Table 2:** National summary data on nine key performance indicators for 121 government hospitals, Ethiopia, 2013

Indicator	Code	Quarter of year				
		First	Second	Third	Fourth	All
**Hospital management**						
Proportion of EHRIG operational standards met, %	KPI 1	70.6	74.7	75.3	77.5	74.5
**Outpatient services**						
Outpatient attendees, No.	KPI 2	586 337	618 442	648 910	648 125	625 453
Outpatient attendees seen by private-wing services, %	KPI 3	7.0	6.6	5.9	6.0	6.4
Outpatient waiting time to treatment, minutes	KPI 4	37.1	40.3	44.9	41.4	41.0
Outpatients not seen on same day, %	KPI 5	0.5	0.5	0.2	0.2	0.3
**Emergency services**						
ED attendees, No.	KPI 6	198 078	203 496	212 982	213 570	828 126
ED patients triaged within 5 minutes of arrival at ED, %	KPI 7	93.6	76.3	94.9	NR	93.0
ED attendees with stay longer than 24 hours, %	KPI 8	2.4	2.1	2.3	2.0	2.2
ED mortality, %	KPI 9	0.3	0.2	0.2	0.2	0.2

ED: emergency department; EHRIG: Ethiopian hospital reform implementation guidelines; KPI: key performance indicator; NR, not reported.

The approach used to establish the Ethiopian system for monitoring hospital performance was designed to fit the Ethiopian context. Many hospital employees were initially unfamiliar with the methods used for reliable and valid data collection and few had adequate experience with computer software. As many of the computers available in hospitals functioned poorly, the system was designed to use relatively simple software programmes.

The main challenges that arose during implementation were errors in data collection and calculation at hospital level and the fear of reprisal for poor performance. For instance, some hospital employees were unsure which denominators or patient populations they should be using. Some hospitals repeatedly failed to report data on particular indicators and some were afraid to report data that highlighted poor performance – especially poor clinical indicators. In the first year of the system, rates of surgical site infection and neonatal mortality were often found to be underreported. Hospitals that appeared to be struggling in reporting reasonably accurate data on the key performance indicators were offered additional on-site training and one-on-one coaching. In their hospital-wide meetings, hospital chief executive officers were encouraged to cultivate an accountable but non-punitive environment. Regional health bureaus reinforced the importance of the data-collection efforts and, by improving the timeliness of their feedback on the summary data to hospitals, helped prompt more immediate exploration and correction of data errors.

The costs of the monitoring system were originally covered by a grant from the United States CDC. Implementing partners were unable to quantify such costs accurately or to separate them from those of other programmatic activities. In addition to the efforts of the nongovernmental organization and university partners, the ministry of health and regional health bureaus made both financial and in-kind contributions to the establishment and maintenance of the monitoring system. Future efforts would benefit from a more explicit analysis of costs.

### Impact of monitoring

As the national monitoring system was fully implemented, rates of hospital reporting of performance indicators increased. This trend indicated changes in hospital functioning and encouraged improvements in performance. In September 2011, 40% of the 114 government hospitals then in Ethiopia were regularly reporting their performance in terms of all 36 key indicators; by September 2013 this had risen to 78%, and by September 2014, 84%.^33^^,^^35^^,^^36^ The collection and analysis of performance data reportedly motivated hospital-based performance-improvement projects – e.g. the introduction of hourly nurse rounding, distinct staff uniforms, continuous pharmaceutical stock reporting and outpatient appointment systems. Between 2012 and 2013, mean adherence to the operational standards increased from 68.2% to 74.5% while the mean number of deliveries attended each month increased from 12 187 to 16 001.^35^^,^^36^

The national monitoring system also improved evidence-based decision-making at both hospital and government level. Comparative performance results were presented at quarterly meetings with hospitals and regional health bureau staff and this allowed for the open review of performance results, feedback and problem solving. Managers at all levels of the health sector aimed to sustain the enthusiasm for performance monitoring. This required continuous investment in the use of data for tangible improvements, media attention and team and organizational rewards and may, in the long term, include institutional accreditation by national bodies. One example was Ethiopia’s recent integration of the 36 performance indicators into a national quality campaign: the Ethiopian Hospital Alliance for Quality. In 2012, the alliance financially rewarded the 15 hospitals that, according to the relevant performance indicators, offered the most positive patient experiences – with about 55 000 United States dollars each. In 2014, the ministry of health began the alliance’s second cycle and prioritized institutional maternal mortality.

## General observations

Our five-year experience of the development and implementation of a national system for monitoring hospital performance led to several key observations. First, technical investment was critical throughout the process. Many hours of research, writing and development of guidelines were needed to develop a core set of performance indicators that were evidence-based, comprehensive but not overwhelming, and precisely described to allow their consistent calculation and reporting. Ethiopia’s ministry of health led the initiative between 2009 and 2014 and now has full operational responsibility. The ministry has a department exclusively charged with overseeing the country’s management of hospital performance – with support from key champions, including the Minister of Health.

Second, while technical support was critical in the development of the indicators and related documentation, political support was paramount to successful implementation. The ministry of health set a consistent direction and held partners accountable to deliver on its vision for quality improvement. The regional health bureaus also demonstrated strong leadership in advocating for additional performance indicators that fit their regional needs and ensured government and hospital ownership of the monitoring system. Although disagreement emerged, senior government officials continued discussions until a negotiated consensus brought a stable solution that all parties could then support. The process of identifying the best key indicators conferred momentum and helped sustain the monitoring efforts. Although such characteristics may be key to making lasting changes, they can be challenging to embed in any large-scale national efforts.^37^

Lastly, both the technical and political inputs were accomplished because of the ability to leverage strong management capacity – which was built at hospital level and supported by the executive master’s degree programme. The importance of management capacity has been highlighted by many studies.^21^^,^^24^^,^^32^^,^^38^^–^^48^ The chief-executive-officer model – i.e. the establishment of a dedicated, qualified person in each hospital who is trained in hospital management and supported by a hospital governing board – was pivotal in the successful implementation of the system for monitoring performance. Without the management capacity provided by this model, the ideas and strategies written in technical and political arenas would not have been translated into practice at the hospital level. Once adequate management capacity has been built, performance management and reporting become achievable – and even desirable for facility-level staff who wish to assess their own progress. The combination of leverage from existing hospital management capacity, technical inputs and political support provided the conditions and tools needed to enable success in this country-led effort to elevate the performance of hospitals in Ethiopia.

## Conclusion

Ethiopia’s implementation of a national system for monitoring hospital performance serves as an example of a low-income country that aims to improve health service delivery via the creation of a culture of accountability. A limitation of our study is that we lacked outcome data and thus were unable to evaluate the impact of the monitoring system on population health. Such an evaluation would require a long and comprehensive follow-up of patients. Despite this limitation, our observations may be helpful to other low-income countries that are seeking to improve the quality of their hospital care. We offer several recommendations. First, a thorough assessment of the health sector’s readiness to change and desire to prioritize hospital quality can be helpful in the early stages of design and implementation. Such an assessment may include interviews with key informants, collection of data about health facilities and human resources and investigation of local university capacity to offer academic programmes in health-care management. Second, partner and donor alignment with the government’s national vision for quality improvement can enhance acceptability and political support. This alignment can enable resources to be focused strategically towards one national effort – rather than be diluted across dozens of potentially competing projects. Finally, early phases of implementation benefit from having modest early goals and the facility for continuous modification and improvement to the performance monitoring system, through active engagement with all stakeholders.
